# The Direct Cooling of the Preoptic-Hypothalamic Area Elicits the Release of Thyroid Stimulating Hormone during Wakefulness but Not during REM Sleep

**DOI:** 10.1371/journal.pone.0087793

**Published:** 2014-02-03

**Authors:** Davide Martelli, Marco Luppi, Matteo Cerri, Domenico Tupone, Marco Mastrotto, Emanuele Perez, Giovanni Zamboni, Roberto Amici

**Affiliations:** 1 Department of Biomedical and NeuroMotor Sciences, Alma Mater Studiorum - University of Bologna, Bologna, Italy; 2 Systems Neurophysiology Division, Florey Institute of Neuroscience and Mental Health, University of Melbourne, Parkville, Victoria, Australia; 3 Department of Neurological Surgery, Oregon Health and Science University, Portland, Oregon, United States of America; 4 Department of Cellular and Molecular Physiology and Center for Neurodegeneration and Repair, Yale University School of Medicine, New Haven, Connecticut, United States of America; St. Joseph's Hospital and Medical Center, United States of America

## Abstract

Thermoregulatory responses to temperature changes are not operant during REM sleep (REMS), but fully operant in non-REM sleep and wakefulness. The specificity of the relationship between REMS and the impairment of thermoregulation was tested by eliciting the reflex release of Thyrotropin Releasing Hormone (TRH), which is integrated at hypothalamic level. By inducing the sequential secretion of Thyroid Stimulating Hormone (TSH) and Thyroid Hormone, TRH intervenes in the regulation of obligatory and non-shivering thermogenesis. Experiments were performed on male albino rats implanted with epidural electrodes for EEG recording and 2 silver-copper wire thermodes, bilaterally placed in the preoptic-hypothalamic area (POA) and connected to small thermoelectric heat pumps driven by a low-voltage high current DC power supply. In preliminary experiments, a thermistor was added in order to measure hypothalamic temperature. The activation of TRH hypophysiotropic neurons by the thermode cooling of POA was indirectly assessed, in conditions in which thermoregulation was either fully operant (wakefulness) or not operant (REMS), by a radioimmunoassay determination of plasmatic levels of TSH. Different POA cooling were performed for 120 s or 40 s at current intensities of 80 mA and 125 mA, respectively. At both current intensities, POA cooling elicited, with respect to control values (no cooling current), a significant increase in plasmatic TSH levels in wakefulness, but not during REMS. These results confirm the inactivation of POA thermal sensitivity during REMS and show, for the first time, that this inactivation concerns also the fundamental endocrine control of non-shivering thermogenesis.

## Introduction

A large body of recently reviewed experimental evidence [1] has shown that, in many homeotherms, somatic and autonomic responses to thermal challenges are fully operant during wakefulness and non-REM sleep (NREMS), but are impaired or inadequate during REM sleep (REMS). The central origin of this thermoregulatory impairment was suggested by the finding that the direct warming or cooling of the preoptic-hypothalamic area (POA), the most important integrative centre for thermoregulation [2], elicited an appropriate thermal response during waking and NREMS, but were ineffective during REMS [3]–[5]. This suspension of POA thermal sensitivity and the view of a brainstem origin of REMS [6] led to the more general hypothesis that the origin of both the autonomic imbalance and the thermoregulatory impairment characterizing this sleep stage resulted from a suspension of hypothalamic integrative activity [1].

This hypothesis has recently been tested by assessing the hypothalamic control of osmoregulation in wakefulness and sleep through the determination of antidiuretic hormone (ADH) release following a central osmotic stimulation [7]. With this approach, the elicitation of a neuroendocrine reflex was used to study a central response to a specific stimulation carried out during behavioral states. This allowed the researchers to explore an endocrine response without taking into account the duration of its peripheral effects. The results showed that the osmotically stimulated secretion of ADH did not differ during the whole wake-sleep cycle. This suggests that the hypothesis that the thermoregulatory impairment characterizing REMS is the result of a general loss of hypothalamic integrative activity [1] should be reconsidered. Since the effectors of thermoregulation, which are non-specific, perform either thermal or non-thermal tasks [8], an alternative hypothesis may be that it is the autonomic imbalance characterizing REMS to be caused by the inactivation of the thermal responsiveness of POA.

In order to further explore the issue of the specificity of the relationship between REMS and the impairment of hypothalamic control of thermoregulation, we sought to determine how a neuroendocrine reflex, closely related to thermoregulation, would respond to a specific elicitation during REMS. Along these lines, we studied the intervention of Thyrotropin-Releasing-Hormone (TRH) neurons in the regulation of thyroid hormone (TH) secretion following a direct cooling of the POA, a stimulus that has previously been proven to be effective in provoking TH secretion in different mammals [9], [10]. In the rat, hypophysiotropic TRH neurons belong almost entirely to the parvocellular division of the hypothalamic paraventricular nucleus (PVN) [11], a cell population which receives inputs from POA [12] and is intermingled with the magnocellular counterpart secreting ADH. In homeotherms, TH intervenes in the maintenance of body temperature by regulating thermogenetic mechanisms belonging to either obligatory or facultative thermogenesis [13]. Obligatory thermogenesis refers to metabolic activity sustaining basal vital functions, while facultative thermogenesis is involved in the adaptation to changes in ambient temperature (Ta). Also, TH intervenes in the metabolic adaptation to low Ta by stimulating the processes of non-shivering thermogenesis (NST) [13]. As a measure of the activation of TRH hypophysiotropic neurons, we determined the changes in the plasmatic levels of TSH following POA cooling.

## Methods

Adult male Sprague-Dawley rats (n = 39, Charles River, weight: 290–310 g), with free access to food and water, kept at 23.0±0.5°C Ta under a 12-h:12-h light-dark (L-D) cycle (L: 9:00 am – 9:00 pm; 100 lux at cage level) were used. The experiments were performed with the approval of the Ethical-Scientific Committee of the University of Bologna, in accordance with the Italian law translation of the European Union Directive (86/609/EEC) and were supervised by the Central Veterinary Service of the University of Bologna and the National Health Authority. All possible measures were taken to ensure that animal pain and discomfort was minimized. Animals were implanted under general anaesthesia (Diazepam 5 mg/kg, i.m.; Ketamine 100 mg/kg, i.p.) with epidural electrodes for EEG recording and with a 70%–30% silver-copper wire thermode (Ø = 0.6 mm); tips placed bilaterally to the POA according to standard atlas coordinates (from Bregma: 0,3 mm posterior; 1,8 mm lateral; 8,4 mm ventral) [14]. Animals were allowed to recover from surgery for at least one week in the thermoregulated and sound-attenuated room where experiments were being carried out. EEG signals were amplified, filtered (high-pass filter: –40 dB at 0.35 Hz; low-pass filter: –6 dB at 35 Hz) and digitalized (sampling rate: 128 Hz). EEG power values were obtained for 4-s epochs by Fast Fourier Transform (FFT) in the Delta (0.75–4 Hz), Theta (5.5–9 Hz), and Sigma (11–16 Hz) bands. Animal behavior was monitored by a video camera. The wake-sleep states, during which the thermal stimulation would be delivered, were selected by two experimenters on the basis of the raw EEG trace, EEG power values and the video camera images. The thermode was cooled by means of two parallel thermoelectric heat-pumps (6×6 mm, Melcor) fixed on an aluminium holder, acting as a heat sink and fed by a low-voltage high-current DC power supply (Sorensen). The cooling system was tested by means of an infrared thermocamera (FLIR) before and after each experimental session.

**Figure 1:**
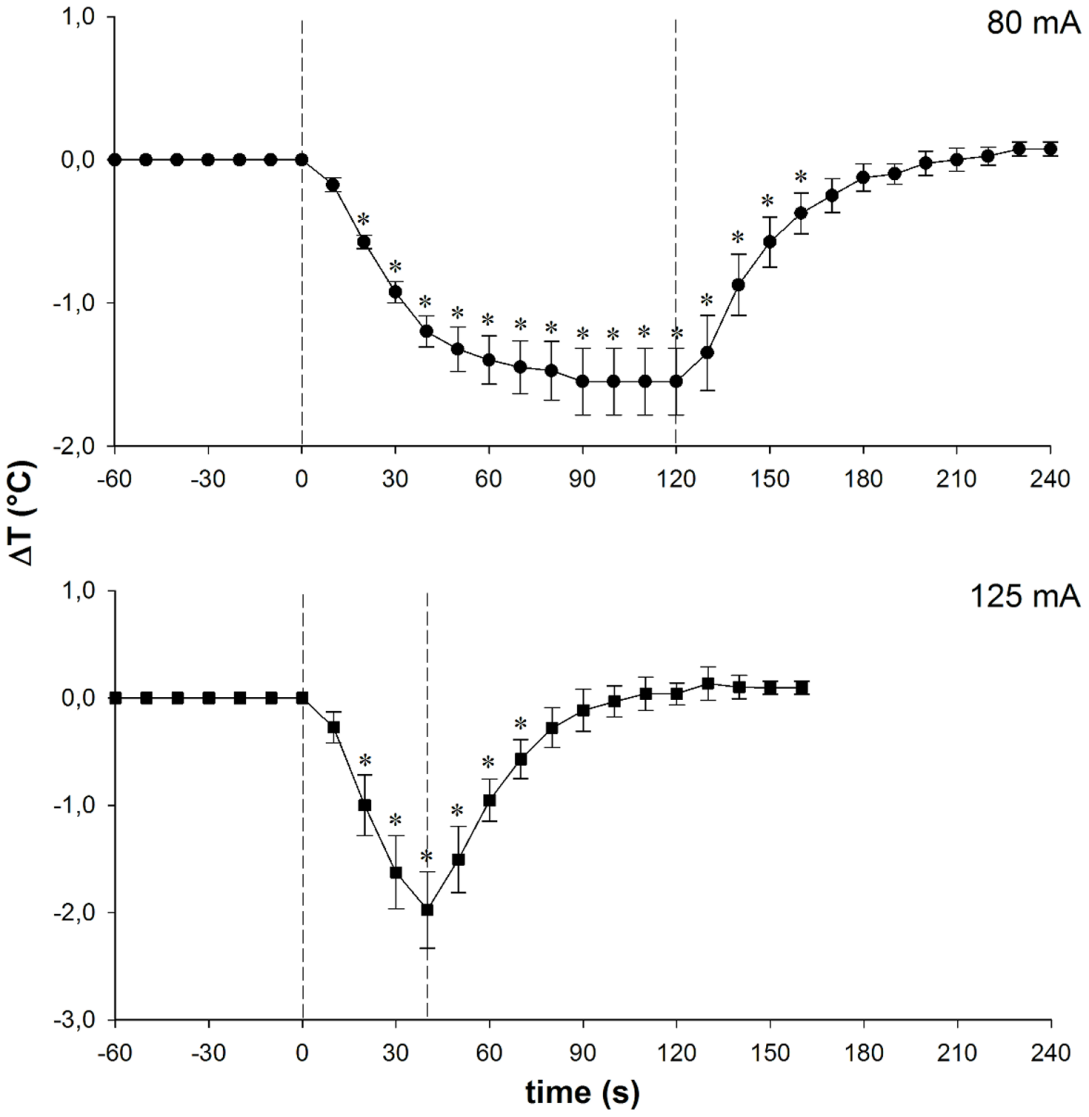
Temperature changes in the preoptic-hypothalamic area elicited by local cooling . Values (°C; means ± S.E.M. from 4 independent observations for each current intensity) are differences from the average baseline levels in the 60 s preceding the onset of cooling. Heat pumps were driven by current intensities of 80 mA (top) and 125 mA (bottom). Asterisks indicate a significant difference from the baseline value at time 0.

Preliminary experiments were carried out in order to determine the following stimulation parameters: A) The highest intensity and longest duration, within the limits of the average REMS time, applicable without awakening animals; in this experiment 5 rats were implanted as described above. The results showed that animals did not wake up when exposed to an 80 mA current for 120 s, while the 125 mA stimulation started to cause animals to wake after 45 s. On these bases, experiments were performed using either 80 mA for 120 s or 125 mA for 40 s. B) Changes in POA temperature (Thy) induced by different current intensities and durations (80 mA for 120 s, 125 mA for 40 s) in 8 anesthetized animals (4 animals for each kind of stimulation) carrying a thermistor (Betatherm) placed 1 mm medially to one of the thermode tips. The results of part B) of the preliminary experiment are shown in Fig. 1: i) 80 mA caused Thy to decrease in about 90 s by 1.5°C, a level that was maintained throughout the rest of the stimulation; Thy re-attained baseline levels in about 45 s; ii) in the 125 mA stimulation, Thy decreased more rapidly (about 40 s) by 2.0°C and re-attained baseline levels in about 35 s.

Experiments were organized according to a 2-factor design. Factors were: A) behavioral state, with REMS and Wake levels; B) stimulation, with control, 80 mA and 125 mA levels. Since the activation of hypothalamic hypophysiotropic neurons was not assessed by monitoring the primary secretion of TRH, but by monitoring its secondary effect on the hypophyseal release of TSH into the general circulation, we had to take into account 2 variables affecting TSH release: i) the time course of the TRH effects; ii) the negative feedback caused by circulating TH. There are no data in the literature concerning TSH release following a direct cooling of POA. However, it has been shown in the rat that: i) plasma TSH concentration is significantly increased 2 min after a single challenge of TRH administered through the jugular vein [15], an interval which is very close to the average duration of REMS episodes in this species [16]; ii) TH exerts a negative feedback on TSH release that shows a latency of about 30 min [17], while its half life ranges from 6 h to 24 h [18].

On the basis of these observations, we decided to: i) avoid the influence that TH would very likely exert on TSH release in a repeated measure factorial design, by using a completely randomized design; ii) promote a larger release of TSH by capitalizing on the time course of the effects of TH on both the release and feedback inhibition of TSH; to this end, we performed POA cooling in two identical consecutive states, separated by a 16±2 min interval from the end of the first stimulation; this interval was calculated considering the plasma half-life of TSH observed in the rat either for the endogenous hormone [19] or following the exogenous administration of a radiolabelled bolus [20].

Experiments started at light onset (9.00 AM). In order to increase sleep propensity, animals were kept awake for 2 hours by a gentle handling performed by the experimenter that manipulated them (weighing, cage and bedding change) during the period spent in the animal house and the recovery following surgery. Cooling stimulations started following the completion of at least one entire wake-sleep cycle and not later than the completion of three. This time lag lasted no more than 1 h. The 6 combinations of the levels of the experimental factors A (Wake and REMS) and B (control, 80 mA and 125 mA) were applied to 6 groups, each of 5 animals. Animals were stimulated in Wake and REMS with either 80 mA or 125 mA; stimuli started 15–20 s from the beginning of each wake-sleep episode and the 4 experimental groups were: Wake80, Wake125, REMS80; REMS125. Consecutive behavioural states were obtained as follows: i) the occurrence of REMS episodes was triggered by a dark pulse (DP, 3 min of lights off) administered during a NREMS episode; DP is known to be very effective in inducing a quick occurrence of consolidated REMS episodes in the albino rat [21]; ii) following the first DP, the light was turned on at the end of cold stimulation, animals were awakened from REMS, and the cycle was repeated. DPs were used in two consecutive Wake episodes, which were obtained through gentle handling; latencies between two consecutive stimuli were precisely matched to those of the stimulated REMS group. Control animals (groups: REMS0, Wake0) underwent the same experimental procedures used for stimulating REMS and Wake groups without switching on the DC power supply (no cooling current).

Since the success rate of REMS induction following a DP administration in NREMS is, at normal laboratory Ta, close to 90% [21], it was virtually impossible to reiterate POA cooling in NREMS within the temporal limits set by the negative feedback of TH and by the TSH half-life. However, since the main aim of the study was to assess the response of the efferent arm of the chosen neuroendocrine reflex during the suspension of POA thermal sensitivity in REMS, we felt that quiet wakefulness would constitute an appropriate control; this was on the basis of the fact that it is firmly established that POA thermoregulatory activity is the same in quiet wakefulness and NREMS [1].

After the end of the double POA cooling, the light was turned on, animals were kept awake for 3 min and then sacrificed by decapitation. The time of the sacrifice of the REMS0 and Wake0 groups was matched with that of the experimental groups in order to avoid any circadian influence on the results. Following decapitation, blood was collected from the trunk in a 50 ml conical tube (Falcon) containing ethylenediaminetetraacetic acid (EDTA), a protease inhibitor cocktail at a final concentration of 1 mg/ml of blood and 0.4 unit/ml of trypsin inhibitor (Sigma). Plasma was separated by centrifugation (3000xg, 15 min, 4°C) and frozen at –80°C. Plasmatic TSH concentration was determined by means of a commercially available radioimmunoassay kit (Amersham Biosciences), using [125I]-TSH as a radioligand tracer and a primary rabbit antiserum. The assay incubation was terminated by adding a solution of polymeric particles coated with donkey anti-rabbit serum and by cold (4°C) centrifugation (1500xg for 10 min). Sediments were washed with 1 mL of phosphate-buffer (0.02 M, pH = 7.65) and tubes were put in a counting vial to which 9 mL of a liquid scintillation cocktail (Packard) was added to determine Auger emission by means of a scintillation counter (Beckman).

Statistical analysis was performed by means of a two-way ANOVA (SPSS 9.0) and significance levels were pre-set at P<0.05. Individual post-hoc comparisons were assessed by means of the modified t-statistics (t*) and significance levels were corrected according to Bonferroni’s sequential method as previously described [7].

## Results

**Figure 2:**
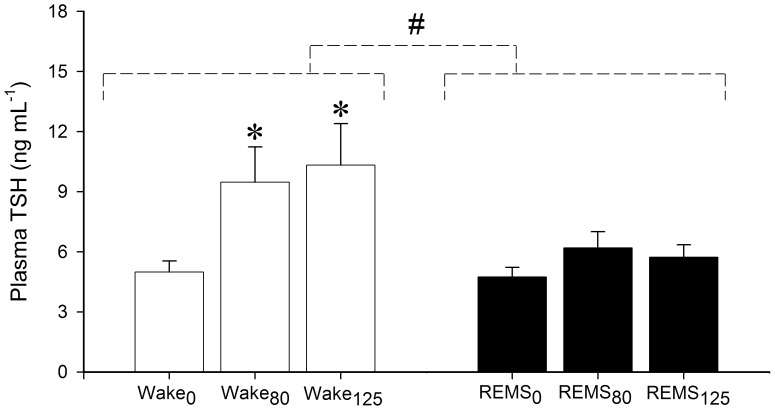
Thyroid Stimulating Hormone concentration in plasma following preoptic-hypothalamic area cooling in behavioral states. Values (ng/mL; means ± S.E.M) are shown for wakefulness (white bars, Wake) and REM sleep (black bars, REMS). Suffixes are: 0, control (no cooling current); 80, 125, cooling current of 80 mA and 125 mA, respectively. # Significant difference between behavioral states. * Significant difference from control.

Changes in plasmatic levels of TSH are shown in Fig. 2. The results show that POA cooling raises the plasmatic concentration of TSH to a level that is higher in wakefulness than in REMS. The statistical analysis showed that the main effects of behavioral state and stimulation factors were significant (factor A, behavioral state: F = 7.353, d.f. 1, P = 0.012; factor B, stimulation: F = 4.224, d.f. 2, P = 0.027), while their interaction was not (F = 1.657, d.f. 2, P = 0.212). The analysis of orthogonal contrasts showed that, with respect to the correspondent controls (Wake0: 5.0±0.6; REMS0: 4.7±0.5), the average stimulated TSH concentration (ng/mL) was: i) significantly different (P<0.05) for stimulated wakefulness (Wake80: 9.5±1.8; Wake125: 10.3±2.1); ii) not significantly different for stimulated REMS (REMS80: 6.2±0.8; REMS125: 5.7±0.6). A further analysis of the stimulation differences showed that TSH concentrations in both (Wake80) and (REMS80) were not significantly different from the correspondent values observed in (Wake125) and (REMS125). Finally, the analysis of individual post-hoc comparisons showed that TSH levels in Wake80 and Wake125 were significantly different (P<0.05, for both) from those in controls (Wake0), while TSH levels in REMS80 and REMS125 were not statistically different from those in controls (REMS0).

The results concerning TSH level following cold stimulation in REMS require further considerations. In fact, although all stimulations ended within the duration of this sleep episode, all the stimulated animals awoke from REMS during the period of re-attainment of Thy baseline levels. Since this introduced variable amounts of wakefulness in a period during which Thy levels were still significantly lower than those of the baseline (Fig. 1), it may be deduced that TSH release had been positively affected by the interaction between the low Thy and the recovery of POA thermal sensitivity. The potential influence of this effect was estimated by calculating, from all the experimental results concerning 80 mA and 125 mA stimulations, the linear regression between the percentage of wakefulness present in the period of time during which Thy was significantly different from the baseline (independent variable) and the values of the plasmatic concentration of TSH (dependent variable): the results gave a significant linear correlation coefficient (R = 0.560, d.f. 18, P<0.05) and the following linear regression equation: y = 0.051x + 4.746. The average percentage of waking during the period of time in which Thy was significantly different from the baseline resulted as 25.2±4.9, a value corresponding to a TSH concentration of 6.03±1,2 ng/mL. This value is very close to the respective average values of REMS80 and REMS125 (Fig. 2).

## Discussion

To the best of our knowledge, the results of the present study are the first to show that the suspension of POA thermal sensitivity characterizing REMS also concerns the endocrine regulation of NST by TH. This has been assessed through changes in TSH release following a short-term and direct POA cooling: a large and significant increase in TSH release follows POA cooling during Wake, but not during REMS. In fact, the small increase in TSH release following POA cooling during REMS, which can explain for the lack of a significant interaction between the "behavioral state" and the "stimulation" factors, appears as fully determined by the awakenings from REMS observed during the re-attainment of the baseline values of Thy after the end of POA stimulation. In this situation animals had a certain amount of wakefulness while Thy levels were still below baseline levels and, thus, able to induce some release of TSH.

Also, the results of this study add a new mammalian species to those in which POA thermal sensitivity during REMS has been addressed before [3]–[5]. However, differently from the previous work, our observations did not concern a direct measurement of the effect of NST activation by POA cooling. This is due to the fact that the serial TRH-TSH-TH secretion affects NST by acting on the brown adipose tissue (BAT) [13] with a longer time course than that induced by the activation of BAT through the descending premotor sympathetic pathways [2] and was assessed, in previous studies [4], [5], by the determination of oxygen consumption.

Our experimental approach appears to minimize the possibility of the intervention of non-specific influences in determining the observed changes in TSH concentration. Firstly, the intervention of handling, although performed without any restraint and by an experimenter to whom animals were accustomed, may be ruled out by the observation that acute immobilization reduces the plasmatic concentration of TSH in the rat [22]. Secondly, the possibility of an inhibition of TRH activity, extending to REMS from the preceding NREMS and induced by either some unknown mechanism or by the short photic stimulation caused by DP may be ruled out due to the equivalence of the basal TSH levels in Wake0 and REMS0. Also, the study of the circadian profile of hormone secretion in the rat showed that a strong surge in TSH plasmatic levels is coincident with the onset of light [23].

A general outcome of these results is the confirmation that the occurrence of REMS is characterized by an inactivation of POA thermal sensitivity, affecting not only the short term somatic adjustments of shivering thermogenesis and evaporative thermolysis [1] but also the fundamental endocrine control of NST. For mammals of a small size the consequences of this outcome may be relevant. For example, when Ta is moved below the inferior limit of thermoneutrality, the rat not only immediately decreases REMS occurrence in a very precise proportion to Ta levels [24], but also shows some adaptive mechanisms which act on a longer term basis: when kept at Ta 0°C for 48 h, animals increase, in the second 24 h period, REMS occurrence well above the low levels reached in the first 24 h period [16]. A similar kind of adaptation is shown by the golden hamster that, following its acclimation to 0°C, displays a daily amount of REMS very close to that normally expressed at thermoneutrality [25]. Since NST is the adaptational response to cold exposure and the basic tool leading to acclimation, it is likely that a return to a REMS occurrence at low Ta towards the levels of thermoneutrality can be explained by the intervention of NST. This possibility is not in contradiction with the results of this work. In fact, it may be hypothesized that the effects of a suspension of the tonic activation of NST during REMS at low Ta is easily overcome by the short duration of the total 24 h REMS time (in the rat at thermoneutrality the maximum amount is about 8–10%) and by the fact that the half-life of TH exceeds the duration of sleep stages [18]. This implies that, in spite of the REMS suspension of POA thermal sensitivity, plasmatic TH concentration may be kept at an almost constant high level during cold exposure. This and the long-lasting peripheral effects of TH may drive the metabolic changes of NST to become much less dependent on the lack of POA thermoregulatory control during REMS episodes. Such a difference between the duration of POA thermal insensitivity and that of the overall control of NST may explain some observations made at low Ta, which show that NST is maintained throughout REMS [26], [27] and BAT temperature significantly decreases during REMS compared to waking and NREMS [28].

In conclusion, it may be hypothesized that the suspension of POA thermal sensitivity is related to some form of central regulation that is negatively affected by a continuous input of thermal peripheral information. This is why POA thermal insensitivity appears to be a pre-requisite of REMS occurrence. The possibility that this process is a local expression of an activity of repair or recovery carried out during sleep on some nervous structures [29] is supported by the observations that a forced long-term deprivation of REMS induces in the rat: i) a life-threatening syndrome characterized by an increase in energy expenditure, a progressive hypothermia and an increase in food intake [30]; ii) specific hypothalamic changes in the expression of peptides involved in the regulation of food intake [31].
